# A framework for enhancing ethical genomic research with Indigenous communities

**DOI:** 10.1038/s41467-018-05188-3

**Published:** 2018-07-27

**Authors:** Katrina G. Claw, Matthew Z. Anderson, Rene L. Begay, Krystal S. Tsosie, Keolu Fox, Nanibaa’ A. Garrison, Summer internship for INdigenous peoples in Genomics (SING) Consortium, Alyssa C. Bader, Jessica Bardill, Deborah A. Bolnick, Jada Brooks, Anna Cordova, Ripan S. Malhi, Nathan Nakatsuka, Angela Neller, Jennifer A. Raff, Jamie Singson, Kim TallBear, Tada Vargas, Joseph M. Yracheta

**Affiliations:** 1 Department of Pharmaceutics, University of Washington, 1959 NE Pacific Street, Seattle, 98195 WA USA; 2 Department of Microbiology, The Ohio State University, 484 W. 12th Ave, Columbus, 43210 OH USA; 3 Department of Microbial Infection and Immunity, The Ohio State University, 460 West 12th Avenue, Columbus, 43210 OH USA; 4 Cardiovascular Institute, University of Colorado Anschutz Medical Campus, 12700 E 19th Ave, Aurora, 80045 CO USA; 5 Interdisciplinary Graduate Program, Vanderbilt University, Nashville, 37235 TN USA; 6 Department of Natural Sciences, Turtle Mountain Community College, PO Box 340, Belcourt, 58316 ND USA; 7 Department of Endocrinology and Metabolic Disease, University of California San Diego, 9500 Gilman Drive, San Diego, 92037 CA USA; 8 Treuman Katz Center for Pediatric Bioethics, Seattle Children’s Hospital and Research Institute, 1900 Ninth Avenue, Seattle, 98101 WA USA; 9 Department of Pediatrics, University of Washington, Seattle, 98101 WA USA; 10 Department of Anthropology, University of Illinois, 607 S. Mathews Ave, Urbana, 61801 IL USA; 11 Department of English, Concordia University, 1455 de Maisonnueve Blvd. W., Montréal, H3G 1M8 QC Canada; 12 Department of Anthropology and Population Research Center, University of Texas at Austin, 2201 Speedway Stop C3200, Austin, 78712 TX USA; 13 School of Nursing, University of North Carolina at Chapel Hill, 504 Carrington Hall, CB 7460, Chapel Hill, 27599 NC USA; 14 Department of Anthropology, University of Colorado at Colorado Springs, 1420 Austin Bluffs Pkwy, Colorado Springs, 80918 CO USA; 15 Department of Anthropology, Animal Biology and Carl R. Woese Institute for Genomic Biology, University of Illinois, 1206 West Gregory Drive, Urbana, 61801 IL USA; 16 Department of Genetics, Harvard Medical School, New Research Building, 77 Ave Louis Pasteur, Boston, 02115 MA USA; 17 Wanapum Heritage Center, PO Box D-4, Beverly, 99321 WA USA; 18 Department of Anthropology, University of Kansas, 1415 Jayhawk Blvd, Lawrence, 66045 KS USA; 19 Division of Student Affairs, University of Illinois, 1401 W. Green Street, Urbana, 61801 IL USA; 20 Faculty of Native Studies, University of Alberta, 1-34 Pembina Hall, Edmonton, T6G 28H AB Canada; 21 Math and Science Department, Oglala Lakota College, PO Box 490, Kyle, 57752 SD USA; 22 Missouri Breaks Industries Research, 118 S Willow Street, Eagle Butte, 57625 SD USA; 23 Department of Environmental Health & Engineering, School of Public Health, Johns Hopkins University, 3400 North Charles Street, Baltimore, 21218 MD USA

## Abstract

Integration of genomic technology into healthcare settings establishes new capabilities to predict disease susceptibility and optimize treatment regimes. Yet, Indigenous peoples remain starkly underrepresented in genetic and clinical health research and are unlikely to benefit from such efforts. To foster collaboration with Indigenous communities, we propose six principles for ethical engagement in genomic research: understand existing regulations, foster collaboration, build cultural competency, improve research transparency, support capacity building, and disseminate research findings. Inclusion of underrepresented communities in genomic research has the potential to expand our understanding of genomic influences on health and improve clinical approaches for all populations.

## Abstract (web summary)

Indigenous peoples are still underrepresented in genetic research. Here, the authors propose an ethical framework consisting of six major principles that encourages researchers and Indigenous communities to build strong and equal partnerships to increase trust, engagement and diversity in genomic studies.

## Introduction

The integration of genomics into healthcare has advanced the ability of researchers and clinicians to predict disease susceptibility and optimize treatment regimens that have the potential to reduce the risk of certain diseases. Routine genome sequencing has become possible with the expanded availability and affordability of next-generation sequencing technology. Recent initiatives such as the *All of Us* research program seek to advance health data integration by collecting genetic information and medical and lifestyle histories from one million individuals (https://allofus.nih.gov/). However, Indigenous peoples, including American Indians (AI), Alaska Natives (AN), and Native Hawaiians (NH), remain underrepresented and understudied in genetic and clinical health research^1–3^, despite facing disproportionately higher rates of cardiovascular disease (CVD), cancer, diabetes, and infectious disease compared with non-Hispanic whites^4,5^. Large-scale research consortia such as the eMERGE Network, CSER, and the International HapMap consortia^6–8^ have successfully recruited only few, if any, Indigenous individuals despite attempts to achieve a representative sample. In fact, despite a numerical increase in participants, the percentage of Indigenous people represented in genome-wide association studies worldwide decreased from 0.06% to 0.05% between 2009 and 2016^1,9^. As such, they are less likely to benefit from genomic research seeking to elucidate the biological etiology of disease, which could aid in disease prevention and treatment and reduce future healthcare disparities^10^.

Globally, Indigenous people are underrepresented in genomic research studies for a variety of reasons including researchers’ failure to engage Indigenous communities in ethical and inclusive ways, lack of study transparency, and historical and recent research malpractice^11–13^, all of which have sowed mistrust and justified peoples’ unwillingness to share personal health information, including DNA, with the research community. We briefly describe two examples of research misconduct and lapses in research ethics. First, the Human Genome Diversity Project (HGDP) and subsequent large-scale efforts such as the National Genographic Project began as endeavors to study worldwide human genetic diversity and global migration patterns. However, these projects failed to fully consider the damaging social and political implications to Indigenous communities that, in the case of the HGDP, prompted strong resistance by the Indigenous Peoples’ Council on Biocolonialism (http://www.ipcb.org/) and tarnished future trust in research and researchers^14–16^. Second, the Havasupai Tribe filed a lawsuit against the Arizona Board of Regents over lack of informed consent and a violation of civil rights in addition to unapproved genetic research with DNA samples^17–19^. The case raised awareness of cultural and political sensitivities around what constitutes appropriate research^20^. These two examples have raised concerns about the negative impacts that research harms (e.g., stigmatization, violation of individuals’ rights, lack of benefit, and cultural incongruence) can have on Indigenous communities.

Despite decades of scientific transgressions across the globe, many Indigenous communities worldwide continue to be interested in genetic research. Some Indigenous communities have developed their own policies to promote responsible conduct of research and have created research review boards to implement mechanisms of accountability that position themselves as partners in research. Some voices within biomedical research have advocated for approaches centered on creating active roles for research participants, community consultation, and research transparency with Indigenous communities—themes embedded in a community-based participatory research (CBPR) approach^14,21–23^. In one case, the San people of Southern Africa, who have been a focal point of genetic research for over 60 years (more recently with a focus on whole-genome studies)^24–26^, issued a code of ethics in early 2017 for researchers conducting studies with their communities^27^. This step was prompted by concerns over the use of insulting terminology in reference to the community, failure to communicate findings to the community, direct recruitment without governance authority, and a lack of investment in the community^28,29^. In other cases, research guidelines such as *Te Mata Ira: Guidelines for Genomic Research with Maori* in New Zealand and the *Human Heredity and Health (H3Africa) Guidelines for Community Engagement* in Africa have empowered Indigenous communities to seek positive outcomes from genomic research^30–32^. Furthermore, the Navajo Nation in the United States (US) has begun to develop a strong culturallyinformed genetic research policy in place of an existing 16-year moratorium on genetic research^33^. Importantly, these emerging guidelines arose from active and frequent communication between researchers (including Indigenous researchers) and community members over many years comprised of multiple training workshops, community meetings, and development of digital and print informational materials. Such efforts to bridge the divide between genomic research and Indigenous peoples will likely alleviate tensions and concerns^34^. While these examples highlight the ongoing concerns of global Indigenous communities, we offer potential solutions to achieve greater equity in genetic research.

## The six principles

### Ethical framework

**Fig. 1:**
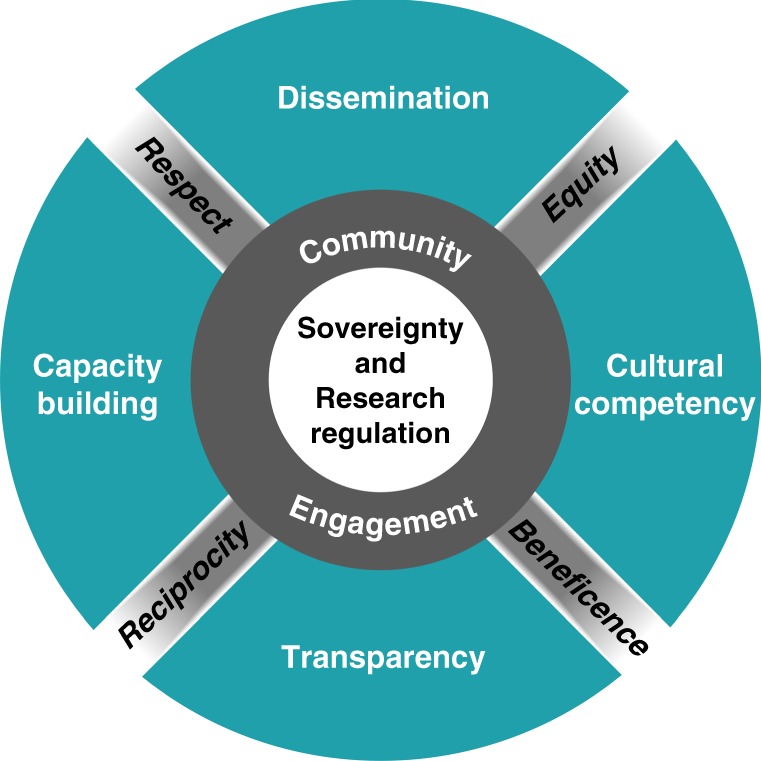
An ethical framework for enhancing genomic research with Indigenous communities. The recognition of tribal sovereignty and research regulations are at the core of our ethical framework. Moving outward from the center, community engagement is necessary to build a foundation to create a collaborative partnership among researchers and community members. Starting from the right going clockwise, researchers should incorporate cultural competency, transparency, capacity building, and dissemination strategies to build trust, increase inclusion of diverse groups in genomic research, and enhance ethical research practices. The diagonal words represent core values that should be used throughout the research process

As Indigenous scientists and allies of the Summer internship for INdigenous peoples in Genomics (SING) Consortium^35^ and also as members of our own tribal communities, we recognize the potential for Indigenous communities to benefit from genomic research. Over the past 2 years, we developed this framework in which we propose the following set of principles (Fig. 1 and Table 1), informed by CBPR approaches, to engage Indigenous people and communities in genomic research. This framework includes six principles: (1) understand existing regulations, (2) foster collaboration, (3) build cultural competency, (4) improve transparency, (5) support capacity, and (6) disseminate research findings. The goals of the framework are to build trust, increase inclusion of diverse groups in genomic research, and enhance ethical research practices that promote tribal research regulations (e.g., tribal oversight and consultation) and benefits to participants and their communities. Our ethical framework extends beyond the current US federal requirements for biomedical and behavioral research (as described by the recently revised Common Rule^36^), which draws upon the principles of bioethics (respect for persons, beneficence, and justice from the Belmont Report), and community-engaged research.

**Table 1:** Principles for engaging in ethical research with Indigenous people

Key considerations	Significance/s	Example/s
*1*. *Understand tribal sovereignty and research regulation* Research oversight structures such as tribal IRBs, the IHS IRB, and tribal council research policies and regulations are important to consider in Indigenous communities.	Tribal sovereignty, respect	Multiple IRB review^43^, NCAI Resource Guide, CRCAIH^52^, NIH Tribal Health Research Office
*2*. *Engage and collaborate with the tribal community* Involve the community in all aspects of the research process from the initial research question development to the final dissemination of results.	Reciprocity	CBPR^45^, Bidirectional knowledge^48^
*3*. *Build cultural competency* Listen and learn from the community about their cultural perspectives.	Respect for persons, traditional knowledge, community values	Cultural competency training^a^
*4*. *Improve transparency of research practices* Make research goals and processes clear and understandable through frequent communication.	Beneficence	Bidirectional knowledge^48^
*5*. *Build tribal research capacity* Train and support community members in the research process.	Tribal sovereignty, beneficence, equity	CRCAIH^52^, NIH Tribal Health Research Office
*6. Disseminate findings in a community-accessible format* Collaborate with community partners to use culturally appropriate methods of disseminating and applying the findings.	Beneficence, respect for persons, equity	Digital storytelling^63^, RED Talks^b^

^a^Tool for Assessing Cultural Competence Training (TACCT): https://www.aamc.org/initiatives/tacct/

^b^RED Talks YouTube Channel: https://www.youtube.com/channel/UCGSdSFOXt5uVK43i67N9-Vg

Although we focus primarily on Indigenous groups of the US (AI, AN, and NH), this framework can inform research practices in many Indigenous communities to incorporate cultural views and acknowledge local research review boards. Few studies have engaged Indigenous communities in genomic research, yet some supportive research collaborations guided by CBPR principles have been ongoing for decades and can serve as examples of what is possible. We illustrate the principles listed below with examples of genetic research with tribes, and highlight how studies have effectively incorporated some of the principles that we present in our ethical framework. Thus, we provide this framework to promote inclusion of historically underrepresented communities in future genomic research, which will expand genomic and health knowledge for diverse populations^37,38^.

### Understand tribal sovereignty and research regulation

Within the US, interactions between researchers and Indigenous people require attention to political considerations as well as the ethical considerations described above. We focus on AI/AN tribes in the US and recognize they are distinct from other underrepresented minorities and global Indigenous groups in that the US government recognizes tribes as domestic-dependent nations and many tribal nations function as sovereign entities (i.e., an autonomous government with the authority to govern and uphold a range of laws including research codes)^39^; however, Native Hawaiians and some US-based tribes remain unrecognized at the state and federal levels^40,41^. In research endeavors involving AI/AN tribes or tribal members, researchers must recognize any existing tribal sovereignty or local governance structures (e.g., the local laws and regulations) before engaging in research^42^. This recognition should be extended to all Indigenous groups regardless of federal or state recognition, as they are distinct and cohesive communities who possess their own social and cultural infrastructures.

Many tribes have regulatory research review structures that may include their community-guided Institutional Review Board (IRB) (e.g., tribal, Indian Health Service (IHS), or healthcare corporation IRB) or tribal councils from which researchers will have to seek approval in addition to their university IRBs. The local review boards (e.g., community IRB or committee) oversee research protocols and may dictate the types of required consent, data and sample usage, and dissemination of research findings in particular communities. Within the US, there are currently 25 IRBs serving tribes and urban Indian communities that are registered with the US Department of Health and Human Services (https://www.ihs.gov/dper/research/hsrp/instreviewboards/). Some IRBs, such as the intertribal Rocky Mountain/Great Plains Tribal Region IRB have created rigorous regulations that promote Indigenous values and community participation for collaborating researchers and serve multiple independent tribes^43^. There is strong support among tribes for “data-sharing procedures that take into account tribal sovereignty and appropriate oversight of research,”^44^ which would be under the purview of the tribal IRBs or research review boards. Policies relating to biospecimen handling and ownership, data storage, and the ultimate return or destruction of samples should be developed early in the research process in collaboration with the local oversight structures.

### Engage and collaborate with the tribal community

To best promote respectful and mutually beneficial research, it is important to recognize the role of historical and current social, political, economic, and environmental influences on health. A comprehensive approach to research that utilizes elements of “community-based research” (also known as community-engaged research) integrates many of these factors^45,46^. Community-based approaches have enhanced research with diverse populations in public health and the social sciences, and the approach has also been endorsed by researchers, funding agencies, and national organizations^47^. Utilizing these approaches, researchers can carefully design studies to build long-term partnerships/collaborations that acknowledge Indigenous peoples’ longstanding knowledge systems and tribal sovereignty (if applicable), account for variables within studies that are not obvious to researchers, and honor Indigenous community values in a culturally respectful manner.

Engaging the community in research can occur at various levels and take many forms. To facilitate community involvement, it is crucial to develop an engagement plan for the research study before its onset and to develop engagement activities for all phases of research. Discussing all aspects of a research study with Indigenous community members and involving them, from the conceptual design to data analysis to the conclusions, can positively impact the research by identifying priority topic areas and questions that are relevant to the community. This, in turn, can generate innovative ideas, more robust hypotheses, and reduce scientific biases. Integrating Indigenous knowledge and perspectives in the research process could be particularly valuable for the analysis, interpretation, and dissemination of the study results.

Building relationships with community members throughout the study increases the likelihood of successfully completing the study and the possibility of developing subsequent long-term partnerships. Collaboration builds trust and can enhance research participation throughout the study’s duration. In some tribal partnerships, one favorable engagement activity has been to implement a tribal advisory council that meets regularly to provide feedback about the ongoing studies and fosters educational opportunities for all participating entities^48^.

The Center for Alaska Native Health Research (CANHR) team incorporated principles of CBPR throughout the initiation and development of their center, with researchers initially generating the questions and then approaching the community partners to determine which questions were of interest^49^. The research questions, study methodology, and dissemination plan were continually refined to reflect community interests through an iterative process of communication and collaboration with a community advisory group (CAG) that ultimately settled on investigations of obesity and diabetes. In particular, the genetic component of the study necessitated the creation of a space for discussing genetic topics between tribal partners and CANHR researchers. The research team utilized the Genetic Education for Native Americans program to conduct a 2-day workshop about basic cell biology, risks and benefits of genetic research, and cultural considerations^50^. The resulting strong collaborative relationship is essential to the longstanding success of the CANHR partnership with tribal communities.

### Build cultural competency

Cultural competency is the ability to acknowledge, communicate, and understand cultural differences while interacting effectively with individuals across cultures. Striving to attain cultural competency is essential for researchers to work toward understanding a community’s cultural values and norms. Researchers should enter Indigenous communities with humility, empathy, and willingness to listen and learn. Without adequate cultural competency in a research project, Indigenous communities may feel that their perspectives and knowledge are devalued or not respected. In particular with biological specimen collection, many Indigenous groups feel that their blood and other tissue samples remain contiguous with the spiritual and physical person throughout their life^51^. In addition, understanding the social, cultural, and structural determinants of health within Indigenous communities (e.g., poverty, barriers to education and employment, and cultural traditions that foster resiliency) is important for contextualizing interactions with the community, creating project objectives, interpreting results, and conducting culturally sustainable science. Therefore, sensitivity to community values, worldviews, restrictions, social circumstances, and the social and economic structures must be practiced when proposing a genomic research study.

Cultural competency also entails working alongside partners with different skills and worldviews. Spending time in the community and being visibly present, through formal research planning meetings and participation in community events, is critical to understand their perspectives, questions, and concerns and for developing relationships. These interactive approaches may allow researchers to identify an appropriate cultural adviser who can aid in navigating the community dynamics. Various resources exist for cultural competency and ethics training for researchers intending to engage with Indigenous communities. There are online and in-person resources for cultural competency training through the Association of American Medical Colleges Tool for Assessing Cultural Competence Training (TACCT). In addition, the National Congress of American Indians (NCAI) AI/AN Genetics Resource Guide (http://genetics.ncai.org) and the Collaborative Research Center for American Indian Health (CRCAIH) offer guidance for researchers to orient themselves to the broader tribal perspective^52^. More recently, the Ethics Training for Health in Indigenous Communities Study (ETHICS) created an alternative training option through the online Collaborative Institutional Training Initiative (CITI) research ethics training course that is tailored for federally-funded researchers working with AI/AN communities^53^. Building a culturally-adept foundation demonstrates respect and enables bidirectional communication, which can further fulfill community needs and provide an indigenized perspective for future research projects.

An example of cultural competency integration in research is found in the Northwest-Alaska Pharmacogenomics Research Network (NWA-PGRN), a collaborative partnership between various research institutions and tribal organizations to study and improve drug response in AI/AN people^54^. The NWA-PGRN established a CAG within each of its participating tribal communities to initiate research on genomic-pharmaceutical drug interactions^48,55^. Central to this collaboration was a bidirectional flow of knowledge: CAG members instructed researchers in cultural competency, traditional knowledge, and science, while researchers conducted workshops and presentations on pharmacogenetics in a community setting (e.g., in the local community rather than at a research institution).

### Improve transparency of research practices

Academic and community goals often differ, necessitating open communication between researchers and community members. To promote transparency, researchers should strive for clarity about the project’s goals in project documents (e.g., research protocol, recruitment materials, consent forms, research results, etc.) and use accessible language. Ideally, the materials should be deliberated upon and approved by community partners and their local review committees before they are disseminated, then revised with feedback as appropriate. Many Indigenous communities may decide to participate in research for the perceived benefits, even if the benefits are not yet realized. As such, researchers must be careful to avoid over-promising benefits from their studies. For example, results that are generated from genomic research often takes years to interpret and disseminate in ways that allow communities to benefit. Even if a new genetic test is produced, it may not offer a health benefit without proper treatment and prevention in place. In addition, if research goals change during the study, it is necessary to keep the community informed. When collected data spurs new hypotheses, the community should be engaged in the formulation of new research directions. To build understanding and trust, community partners can be invited into the laboratory and shown where samples are stored, how they are processed, and how the data are generated. Finally, the anticipated future use of data, samples, and long-term ownership/storage of sequence data must be discussed thoroughly before the study begins. To formalize partnerships, there are various examples of potential agreements such as a Memorandum of Understanding that should be considered by all parties involved and the future disposition of data and samples defined in a Materials Transfer Agreement or Data Sharing Agreement.

Ethical concerns and lack of transparency led some academic institutions to initiate moratoriums on research of archived Indigenous materials, as occurred at the Australian National University in the 1990s. Following this decision, the National Center for Indigenous Genomics (http://ncig.anu.edu.au/) was created in 2013 to regulate archived materials and promote genomic research of benefit to Indigenous Australians. The Center is under Indigenous governance, composed of an Indigenous-majority Board, who ensures Indigenous custodianship of biological samples, proper engagement, insight into research directions and programming, and dissemination of information.

### Build tribal research capacity

Building tribal research capacity by which Indigenous scientists can lead research that is directly aligned with community values is essential for long-term culturally sustainable science. From 2004–2014, the percentages of doctoral degrees awarded annually in science and engineering to Indigenous people in the US was less than 0.4% (in 2014, 110 AI/AN and 33 NH out of 38,939 nationwide were awarded doctoral degrees), which highlights the disparity in the number of trained Indigenous researchers^56,57^. The participation of Indigenous people and other underrepresented minority populations in Science, Technology, Engineering and Mathematics (STEM) fields remains shockingly low, and Indigenous students often face various barriers that preclude them from pursuing or continuing careers in STEM fields^57^, such as inadequate academic preparation, competing family or cultural demands, conflicting epistemologies, income barriers, and poor mentorship. Allies in leadership positions at academic and federal institutions can play an important role in building research capacity to increase training opportunities for Indigenous scientists to conduct health and genomic studies within their communities. Therefore, the research community should actively work to continually train, engage, mentor, and support emerging Indigenous scientists from the trainee level to beyond the early career scientist stage.

Further tensions surrounding genomic research may stem from a disconnect between scientists and the Indigenous communities involved in research. Around the world, social and economic inequities have contributed to the lack of recruitment and inclusion of Indigenous people in genomic studies, education, and training. Indigenous people are more likely to live in locations distant from the universities and institutions conducting the research and thus may have less experience working with researchers. In closing this divide, Indigenous scientists can act as cultural brokers and serve as liaisons between their communities and academic research institutions with a capacity to lead future projects. Ultimately, a critical mass of Indigenous scientists can become self-sustaining, providing beneficial impact within both the broader scientific community and their own communities.

Building research capacity within Indigenous communities strengthens inclusivity in biomedical research and promotes the use of culturally appropriate frameworks. Inclusion of community and Indigenous researchers can bring greater transparency of the research process to Indigenous communities and provide training opportunities for them to become involved in the research. Those seeking guidance beyond local tribal governance may also contact the newly appointed Director of Tribal Health Research Office, which coordinates NIH research related to the health of AI/AN across the NIH Institutes, Centers, and Offices, and recently released a Tribal Consultation Policy (https://dpcpsi.nih.gov/thro).

An example of a study that has built research capacity in tribal communities is the Strong Heart Study (SHS). SHS was founded in 1988 to investigate CVD among 12 participating tribes across the country and stands as the largest epidemiological study to date among AIs and the second longest running study of CVD in the US^58^. Recent investigations focus on identifying genetic risk factors of CVD in participating families recruited from health centers and academic institutions^59–61^. To build trust across these distinct communities, SHS investigators worked closely with community leaders to identify specific questions of interest within the scope of the study and hired community members to work within the study group. Consequently, SHS maintains 90% retention rates across study phases and has incorporated dozens of AI/AN investigators who participate in active research teams, including the training of many Indigenous PhDs, MDs, nurses, and other medical professionals from tribal communities^62^. SHS has served as an important model for collaborative efforts with Indigenous groups.

### Disseminate findings in a community-accessible format

Research findings are usually published in academic journals, yet the publications are rarely accessible to Indigenous communities nor do they tend to enter the community discourse, thus limiting the impact to community knowledge and benefit. Soliciting community input regarding best practices to disseminate research findings to participating and affected communities can reverse this trend. Broadening the format of disseminated research results using creative solutions offers community partners greater access to the materials (e.g., digital storytelling, community newsletters or reports, social media, radio, and small grassroots interventions). For example, rural AN communities have used digital storytelling (culturallyimmersed videos) to share knowledge about health promotion and cancer awareness within their community^63^. Inspired by TED Talks, video-based content has also been developed through RED Talks, an online YouTube channel designed to share solution-based ideas, tribal research and policies, and success stories across tribes. With access to such information, communities can generate tool kits and further policies.

Acknowledging the contributions of community partners is imperative for strengthening partnerships. Researchers can do this by inviting community members who contributed in meaningful ways to the research process to be co-authors or acknowledging their contributions in publications, disseminated materials, or presentations. Lack of acknowledgment actively disengages partner communities from the research process, removing their insight, efforts, and work in the research process. At a minimum, communities should be included in the acknowledgements to indicate their central role in all completed studies. Ownership of the study is thus transferred back to the community while instilling added confidence in future research endeavors.

Our framework considerations are multifaceted and interconnected. Each research study will have different levels of collaboration and be unique in its own way. Depending on the Indigenous community, some research projects may need to apply only a few of the principles outlined above. Ultimately, the study goals and the dynamics of the community–researcher partnership will evolve throughout the duration of the project. Application of these principles, as demonstrated by collaborative genomic research projects such as those between the White Mountain Apache and the National Institutes of Health^64^, or the Maori tribes in New Zealand and the University of Otago (https://www.otago.ac.nz/full-circle/), suggest that research programs between Indigenous people and scientific communities can flourish when commitment and transparency are prioritized.

## Future directions

The suggested framework requires deliberate investments in time and effort by researchers and Indigenous collaborators. Building strong and equal partnerships is necessary for establishing long-lasting successful relationships that have the potential to greatly benefit future research endeavors. Acknowledging the commitment required to incorporate these ethical principles of genetic research in Indigenous communities means new supportive policies and substantial investments from federal agencies and institutions will be necessary. Given that diversity in research is a top priority for many agencies and universities^65,66^, their commitment to this effort must be demonstrated by actions that enable and facilitate our framework for underrepresented communities. Without such policies, entire communities of Indigenous people will continue to be ignored, despite research programs’ best intentions.

Moving forward, genomic research partnerships between academic institutions and Indigenous peoples can be fostered by viewing participating groups as collaborators instead of research subjects. Presently, with the new *All of Us* research program, technological advancements, and increased awareness of enlightened research approaches, we have the opportunity to usher in an era of widespread respectful and beneficial research that has the potential to truly engage Indigenous and other underrepresented populations in genomic research. This framework offers an opportunity for researchers to begin working with Indigenous communities, which provides a richness of discoveries that are both scientifically and ethically motivated. As ethical researchers, we must strive to incorporate the presented framework into the current research paradigm and undertake this challenge of collaborating and engaging with Indigenous communities to address the health challenges faced by these communities. More broadly, other Indigenous communities can choose to use this framework to promote stronger engagement so that the benefits from scientific advances are distributed more meaningfully and equitably.

## References

[1] Popejoy AB, Fullerton SM (2016). Genomics is failing on diversity. Nature.

[2] Jaja C, Burke W, Thummel K, Edwards K, Veenstra DL (2008). Cytochrome p450 enzyme polymorphism frequency in indigenous and native american populations: a systematic review. Community Genet..

[3] Chen MS, Lara PN, Dang JH, Paterniti DA, Kelly K (2014). Twenty years post-NIH Revitalization Act: enhancing minority participation in clinical trials (EMPaCT): laying the groundwork for improving minority clinical trial accrual: renewing the case for enhancing minority participation in cancer clinical trials. Cancer.

[4] Jacobs-Wingo JL (2016). Causes and disparities in death rates among urban American Indian and Alaska native populations, 1999-2009. Am. J. Public Health.

[5] Adekoya N, Truman B, Landen M, Centers for Disease Control and Prevention. (2015). Incidence of notifiable diseases among American Indians/Alaska Natives—United States, 2007-2011. Mmwr. Morb. Mortal. Wkly. Rep..

[6] Gottesman O (2013). The Electronic Medical Records and Genomics (eMERGE) Network: past, present, and future. Genet. Med..

[7] Green RC (2016). Clinical sequencing exploratory research consortium: accelerating evidence-based practice of genomic medicine. Am. J. Hum. Genet..

[8] Frazer KA, International HapMap Consortium (2007). A second generation human haplotype map of over 3.1 million SNPs. Nature.

[9] Bustamante CD, Burchard EG, De la Vega FM (2011). Genomics for the world. Nature.

[10] West KM, Blacksher E, Burke W (2017). Genomics, health disparities, and missed opportunities for the nation’s research agenda. JAMA.

[11] Mello MM, Wolf LE (2010). The Havasupai Indian tribe case–lessons for research involving stored biologic samples. N. Engl. J. Med..

[12] Bowekaty MB, Davis DS (2003). Cultural issues in genetic research with American Indian and Alaskan Native people. IRB.

[13] Dukepoo FC (1998). Genetic services in the new era: Native American perspectives. Community Genet..

[14] Dodson M, Williamson R (1999). Indigenous peoples and the morality of the Human Genome Diversity Project. J. Med. Ethics.

[15] Greely HT (2001). Human genome diversity: what about the other human genome project?. Nat. Rev. Genet..

[16] TallBear K (2007). Narratives of race and indigeneity in the Genographic Project. J. Law Med. Ethics.

[17] *Havasupai Tribe of Havasupai Reservation v. Arizona Board of Regents and Therese Ann Markow*. 2008: Nos. 1 CA-CV 07-0454, 1 CA-CV 07-0801.

[18] Sterling RL (2011). Genetic research among the Havasupai–a cautionary tale. Virtual Mentor..

[19] Harmon, A. Indian Tribe Wins Fight to Limit Research of Its DNA. *The New York Times*. https://www.nytimes.com/2010/04/22/us/22dna.html (April 22, 2010).

[20] Garrison NA, Cho MK (2013). Awareness and acceptable practices: IRB and researcher reflections on the Havasupai Lawsuit. AJOB Prim. Res..

[21] Blumenthal DS (2011). Is community-based participatory research possible?. Am. J. Prev. Med..

[22] Skinner HG (2015). Using community-based participatory research principles to develop more understandable recruitment and informed consent documents in genomic research. PLoS ONE.

[23] Smith LT (1999). 1950-Decolonizing Methodologies: Research and Indigenous Peoples.

[24] Schuster SC (2010). Complete Khoisan and Bantu genomes from southern Africa. Nature.

[25] Nurse GT (1975). A reassessment of the HL-A system in Khoisan populations of South West Africa. Tissue Antigens.

[26] Jenkins T, Lane AB, Nurse GT, Tanaka J (1975). Sero-genetic studies on the G/wi and G//ana San of Botswana. Hum. Hered..

[27] South African San Institute 2017. *San Code of Research Ethics*. (2017).

[28] Nordling, L. *San People of Africa Draft Code of Ethics for Researchers*. 10.1126/science.aal0933 (2017).

[29] Chennells, R. & Steenkamp, A. In: *Ethics Dumping: Case Studies from North-South**Research Collaborations*. (eds Schroeder D., Cook J., Hirsch F., Fenet S. & Muthuswamy V.) 15–22 (Springer International Publishing, Cham, 2018).

[30] Hudson M (2016). Te Mata Ira: Guidelines for Genomic Research with Māori.

[31] A Welcome Framework for Research in Africa. *Nature*. **556**, 274 (2018). 10.1038/d41586-018-04589-0 29670269

[32] Friedrich, M. Ethical guidelines for genomic research in Africa. *JAMA* **319**, 2371 (2018). 10.1001/jama.2018.7241 29922834

[33] Reardon S (2017). Navajo Nation reconsiders ban on genetic research. Nature.

[34] Jacobs B (2010). Bridging the divide between genomic science and indigenous peoples. J. Law Med. Ethics.

[35] Malhi RS, Bader A (2015). Engaging Native Americans in genomics research. Am. Anthropol..

[36] Office of Human Research Protections. Federal Policy for the Protection of Human Subjects. *Federal Register* **82**, 7149-7274 (2017). 28106360

[37] Smith CE (2016). Using genetic technologies to reduce, rather than widen, health disparities. Health Aff. (Millwood).

[38] Reardon S (2015). US tailored-medicine project aims for ethnic balance. Nature.

[39] Kalt, J. P. & Singer, J. W. *Myths and Realities of Tribal Sovereignty: The Law and Economics of Indian Self-Rule.* KSG Faculty Research Working Paper RWP04-016 (Harvard University, 2004).

[40] Dombrowski K (2005). Forgotten tribes: Unrecognized Indians and the federal acknowledgment process. Mark Edwin Miller. J. Anthropol. Res..

[41] Kehaulani, K. J. Precarious positions: Native Hawaiians and US federal recognition. *The Contemporary Pacific* **17**, 1–27 (2005).

[42] Hull SC, Wilson (Diné) DR (2017). Beyond Belmont: Ensuring respect for AI/AN communities through tribal IRBs, laws, and policies. Am. J. Bioeth..

[43] Kelley A, Belcourt-Dittloff A, Belcourt C, Belcourt G (2013). Research ethics and indigenous communities. Am. J. Public Health.

[44] James R (2014). Exploring pathways to trust: a tribal perspective on data sharing. Genet. Med..

[45] Burhansstipanov L, Christopher S, Schumacher SA (2005). Lessons learned from community-based participatory research in Indian country. Cancer Control.

[46] Israel BA, Schulz AJ, Parker EA, Becker AB (1998). Review of community-based research: assessing partnership approaches to improve public health. Annu. Rev. Public Health.

[47] Dankwa-Mullan I (2010). The science of eliminating health disparities: summary and analysis of the NIH summit recommendations. Am. J. Public Health.

[48] Woodahl EL (2014). Pharmacogenetic research in partnership with American Indian and Alaska Native communities. Pharmacogenomics.

[49] Boyer BB (2005). Building a community-based participatory research center to investigate obesity and diabetes in Alaska Natives. Int. J. Circumpolar. Health.

[50] Burhansstipanov L, Bemis L, Dignan M, Dukepoo F (2001). Development of a genetics education workshop curriculum for Native American college and university students. Genetics.

[51] Sahota PC (2014). Body fragmentation: Native American community members’ views on specimen disposition in biomedical/genetics research. AJOB Empir. Bioeth..

[52] Elliott AJ (2015). Fostering social determinants of health transdisciplinary research: The Collaborative Research Center for American Indian Health. Int. J. Environ. Res. Public. Health.

[53] Pearson, C. R., Parker, M., Zhou, C. Donald, C., & Fisher, C. B. A culturally tailored research ethics training curriculum for American Indian and Alaska Native communities: a randomized comparison trial. *Crit. Public Health*10.1080/09581596.2018.1434482 (2018). 10.1080/09581596.2018.1434482 PMC6320230 30613127

[54] Giacomini KM (2007). The pharmacogenetics research network: from SNP discovery to clinical drug response. Clin. Pharmacol. Ther..

[55] Boyer BB (2011). Ethical issues in developing pharmacogenetic research partnerships with American Indigenous communities. Clin. Pharmacol. Ther..

[56] National Center for Science and Engineering Statistics. *Women, Minorities, and Persons with Disabilities in Science and Engineering: 2017*. Special Report NSF 17-310 (National Science Foundation, Arlington, VA, 2017).

[57] James R, Starks H, Segrest VA, Burke W (2012). From leaky pipeline to irrigation system: minority education through the lens of community-based participatory research. Prog. Community Health Partnersh..

[58] Lee ET (1990). The Strong Heart Study. A study of cardiovascular disease in American Indians: design and methods. Am. J. Epidemiol..

[59] North KE (2003). Evidence for distinct genetic effects on obesity and lipid-related CVD risk factors in diabetic compared to nondiabetic American Indians: the Strong Heart Family Study. Diabetes Metab. Res. Rev..

[60] North KE (2003). Genetic and environmental contributions to cardiovascular disease risk in American Indians: the strong heart family study. Am. J. Epidemiol..

[61] North KE (2003). Evidence for genetic factors underlying the insulin resistance syndrome in American Indians. Obes. Res..

[62] Sambo BH (2001). The Strong Heart Study: interaction with and benefit to American Indian communities. Am. J. Med. Sci..

[63] Cueva M, Kuhnley R, Revels L, Schoenberg NE, Dignan M (2015). Digital storytelling: a tool for health promotion and cancer awareness in rural Alaskan communities. Int. J. Circumpolar. Health.

[64] Bliss SJ (2008). Invasive pneumococcal disease among White Mountain Apache adults, 1991-2005. Arch. Intern. Med..

[65] Hurtado S, White-Lewis D, Norris K (2017). Advancing inclusive science and systemic change: the convergence of national aims and institutional goals in implementing and assessing biomedical science training. BMC Proc..

[66] James, S. M. & Singer, S. R. From the NSF: The National Science Foundation’s investments in broadening participation in science, technology, engineering, and mathematics education through research and capacity building. *CBE Life Sci. Educ. Fall*. **15**, fe7 (2016). 10.1187/cbe.16-01-0059 PMC5008905 27587853

